# Diagnostic pathology in 2013: facts, thoughts, and perspectives

**DOI:** 10.1186/1746-1596-9-2

**Published:** 2014-01-09

**Authors:** Klaus Kayser

**Affiliations:** 1 Humbold University Berlin, Charite Platz 1, Berlin, Germany

**Keywords:** Open access publication, Virtual slides, Publication procedure, Diagnosticpathology.org

## Abstract

**Abstract:**

Herein we analyze the development of the open access, peer reviewed journal Diagnostic Pathology (http://www.diagnosticpathology.org) during the past year and try to forecast its perspectives in 2014.

**Virtual slides:**

The virtual slide(s) for this article can be found here: http://www.diagnosticpathology.diagnomx.eu/vs/1562693099114452.

## Facts

The journal increased in its impact factor by 9.5% (from 1.69 to 1.85). The number of submissions and publications grew by a similar percentage, whereas rejections remained constant at 40%. In 2006, when the journal was founded, case reports dominated – yet now largely replaced by research papers. Originally dealing only with human disease, the journal now accepts veterinary research as well as articles investigating therapeutic responses or representing meta-analyses. A broad spectrum of routine research, innovative science, recently implemented new ideas, reviews and letters to the editor are also distributed to readers. The journal site is visited over 50,000 times per month by researchers, mostly from the USA, followed by India and China. Maintaining these themes, we cordially invite authors to submit for publication their scientific ideas, research data, medical diagnostics, and results of theoretical and applied pathology.

## Performance

We try to review all submitted articles in a fair and independent manner. The comments of at least two reviewers are used to accept, modify or reject a submission. Authors are invited to propose reviewers for their paper, and their wishes are normally accepted. We aim for a short turn-around period for submissions, and want urgently to shorten it: currently, the mean period between date of submission and publication is still 70 days. Approximately 50% of invited reviewers do not respond, providing a significant delay in handling authors’ submissions. In turn authors require an excessive time to revise their paper, despite reminders. Consequently, we have had to further reject – reluctantly – some 5% of previously-accepted submissions.

## Electronic submission and handling system

The current submission system uses somewhat outdated technology. Potentially automated steps require interactive (manual) interventions. Important communication between authors, reviewers and editors is not presently monitored, requiring several hours of independent editing daily. The publisher’s concept of placing all its journals under the same umbrella - in terms of science, number of submissions, creativity, and performance - is a constraint towards achieving progress. Our journal however contributes only minimal profit to the publisher. Additional personal elements are also present.

## Financial elements

Authors must pay once their article has been accepted for publication. The fee has been established at the publisher’s discretion, without reference to the editor. The basis of their calculation is not apparent. The present publication charge is 1115 GBP (1315 € or 1780 US$). Despite the considerable increase in published articles in 2013, the publication charge will increase to 1170 GBP (1380 € or 1870 US$). We appreciate that the publisher offers a significant reduction of its publication charge to colleagues working in developing countries. Fees are adjusted according to the official financial situation of the respective country.

## Specificities of *diagnosticpathology.org*

The open access journal site, Diagnostic Pathology is organized in a similar manner to peer-reviewed printed journals. Articles are published and may be cited in a date-oriented citation method, in combination with pseudo-page numbering. This feature permits easy and issue-oriented retrieval as well as simple survey and reading.

Diagnostic Pathology still remains the only scientific journal that offers the publication of virtual slides (VS): i.e. large data images of completely digitized microscopic glass slides. The mandatory logistic background is managed by three digitalization centres which cover America (Huron Inc. Waterloo, Canada), Asia and Africa (Motic Inc., Xiamen, China), and Europe (Leica, Wetzlar, Germany) together with a central logistic manager (Diagnomx Inc., Waltrop, Germany). The publication of virtual slides requires external image servers and viewers, continuous registration of articles and included images (DOI), and a separation of VS processing from article publication, in order to avoid delays in publication. Therefore, dummy links have been implemented which can be replaced by the article’s links at any time and independently from the date of publication.

The article-associated publication of VS offers several advantages. These include:

a) Microscopic image viewing by intellectual distinguishing information content and area of interest (field of view);

b) Comparison of the area of main interest with its environment;

c) Quality evaluation of submitted images;

d) Potential use for automated image analysis and measurements. Applicable systems are already available, with open access examples offered by the Virtual International Pathology Institute (VIPI), (http://www.diagnomx.eu/vipi);

e) Potential source of unique educational and research material in combination with the submitted text.

In 2013, we were able to include approximately 200 VS in some 75 published articles: approximately two thirds of suitable articles have been equipped with VS. We do thank authors for their efforts, support and enthusiasm in taking advantage of the recent development of electronic communication tools in order to further develop the journal.

## Editorial policy

In principle, running peer-reviewed scientific journals should be a compromise between the publisher’s financial interests and the editor’s assurance of quality and information distribution in science and research. The editor should be able to independently define policy and not simply follow the publisher’s decisions: publishers and their administration tend to be motivated mainly by short term profit. Certainly we do have responsibility to the general financial situation of our journal; however, to maximize profit is not one of our aims. Despite this somewhat negative element, we still aim to improve communication in research and science and to promote our journal in this direction.

## Perspectives

Competition between open access journals is increasing. Editors and publishers should be aware that even ‘big players’ will become victims of recently developed electronics and networking if they remain focussed on short term profit - as was the fate of large mobile phone companies. Our targets focus on solving discrepancies in scientific quality, reviewer comments, publication velocity and article documentation and author-reader interaction. Some of these tools will probably be accessible to our readers and authors in the coming year. We aim to test automated review tools, on-line interactive author-reader communication, and continuously open article communication. We will guide authors and readers to new horizons of scientific communication.

Christmas is close. It confers unlimited hope and joyful anticipation, especially for children with their expected numerous gifts. I wholeheartedly wish all our readers, authors, reviewers, and supporters a ‘Merry Christmas’ and a fruitful, healthy, and enjoyable New Year. I also thank all members of the editorial board and I appreciate very much all their support and efforts in keeping our journal running at its present high level. I include myself in joyful anticipation on having you all on board again when the coming New Year starts with its challenges and perspectives.

Klaus Kayser

Editor-in-chief

